# Dual‐energy CT‐based stopping power prediction for dental materials in particle therapy

**DOI:** 10.1002/acm2.13977

**Published:** 2023-04-09

**Authors:** Friderike K. Longarino, Christopher Herpel, Thomas Tessonnier, Stewart Mein, Benjamin Ackermann, Jürgen Debus, Franz Sebastian Schwindling, Wolfram Stiller, Andrea Mairani

**Affiliations:** ^1^ Clinical Cooperation Unit Radiation Oncology German Cancer Research Center (DKFZ) Heidelberg Germany; ^2^ Department of Radiation Oncology Heidelberg University Hospital Heidelberg Germany; ^3^ Department of Physics and Astronomy Heidelberg University Heidelberg Germany; ^4^ Department of Prosthodontics Heidelberg University Hospital Heidelberg Germany; ^5^ Heidelberg Ion Beam Therapy Center (HIT) Heidelberg Germany; ^6^ Translational Radiation Oncology German Cancer Research Center (DKFZ) Heidelberg Germany; ^7^ Heidelberg Institute of Radiation Oncology (HIRO) National Center for Radiation Research in Oncology (NCRO) Heidelberg Germany; ^8^ National Center for Tumor Diseases (NCT) Heidelberg Germany; ^9^ German Cancer Consortium (DKTK) Core Center Heidelberg Heidelberg Germany; ^10^ Diagnostic & Interventional Radiology (DIR) Heidelberg University Hospital Heidelberg Germany; ^11^ Medical Physics National Center of Oncological Hadrontherapy (CNAO) Pavia Italy

**Keywords:** dental materials, dual‐energy CT, dual‐layer spectral CT, particle therapy, range uncertainty, stopping power ratio, treatment planning

## Abstract

Radiotherapy with protons or light ions can offer accurate and precise treatment delivery. Accurate knowledge of the stopping power ratio (SPR) distribution of the tissues in the patient is crucial for improving dose prediction in patients during planning. However, materials of uncertain stoichiometric composition such as dental implant and restoration materials can substantially impair particle therapy treatment planning due to related SPR prediction uncertainties. This study investigated the impact of using dual‐energy computed tomography (DECT) imaging for characterizing and compensating for commonly used dental implant and restoration materials during particle therapy treatment planning. Radiological material parameters of ten common dental materials were determined using two different DECT techniques: sequential acquisition CT (SACT) and dual‐layer spectral CT (DLCT). DECT‐based direct SPR predictions of dental materials via spectral image data were compared to conventional single‐energy CT (SECT)‐based SPR predictions obtained via indirect CT‐number‐to‐SPR conversion. DECT techniques were found overall to reduce uncertainty in SPR predictions in dental implant and restoration materials compared to SECT, although DECT methods showed limitations for materials containing elements of a high atomic number. To assess the influence on treatment planning, an anthropomorphic head phantom with a removable tooth containing lithium disilicate as a dental material was used. The results indicated that both DECT techniques predicted similar ranges for beams unobstructed by dental material in the head phantom. When ion beams passed through the lithium disilicate restoration, DLCT‐based SPR predictions using a projection‐based method showed better agreement with measured reference SPR values (range deviation: 0.2 mm) compared to SECT‐based predictions. DECT‐based SPR prediction may improve the management of certain non‐tissue dental implant and restoration materials and subsequently increase dose prediction accuracy.

## 1 INTRODUCTION

Radiotherapy with protons or light ions can offer accurate and precise treatment delivery to cure tumors.^1^ Head and neck tumors are among the indications for particle therapy, since it is able to reduce the volume of irradiated healthy tissues by more than 25% and thus more effectively spare organs‐at‐risk.^2^ However, dental implant and restoration materials, which often exhibit uncertain stoichiometric composition, can be a source of larger uncertainty in the ion beam range.^2^, ^3^ For example, if metals are crossed by a proton beam, large differences between planned and delivered ranges with deviations up to several millimeters can occur in the patient, resulting in a dose deposition at an unexpected depth along the beam axis.^4^, ^5^ These range uncertainties can result from artifacts of dental implant and restoration materials on computed tomography (CT) treatment planning images for head and neck radiotherapy,^6^ which are mainly caused by beam hardening and photon starvation and degrade the quantitative accuracy of CT numbers,^3^ as well as from the CT‐based estimation of stopping power ratio (SPR).^7^ Since the SPR values are conventionally assigned from a heuristic CT‐number‐to‐SPR conversion, which does not cover non‐tissue implant materials,^8^ errors in the treatment planning system (TPS)‐calculated dose distribution can result. These systematic uncertainties may impact patient outcome in terms of local tumor control or normal tissue toxicity.^7^

To address the uncertainties arising from artifacts on CT images and the conventional CT‐number‐to‐SPR conversion, different imaging techniques have been investigated. Over the past decade, dual‐energy CT (DECT) systems have become increasingly available in the clinic. DECT generates image data from two x‐ray acquisitions using differing energy ranges. It thus increases the available quantitative data and possibilities for material characterization compared to classical single‐energy CT (SECT). Several different technical approaches for acquiring DECT image data have emerged with unique features and compromises to be balanced for each application,^9^ comprising dual‐source CT, sequential acquisition CT (SACT), twin‐beam CT, fast kVp‐switching CT, and dual‐layer spectral CT (DLCT).^8^, ^9^, ^10^, ^11^ In various studies conducted with tissue surrogates or biological tissues as well as in patient analyses, DECT showed improved SPR prediction for particle therapy compared to SECT.^8^, ^12^, ^13^, ^14^, ^15^, ^16^, ^17^, ^18^ Besides human tissue, the usage of DECT in particle therapy planning may also be advantageous for non‐tissue implant materials.^12^, ^18^

For treating patients with dental implant and restoration materials, different avoidance strategies have been employed so far. For example, non‐ideal beam geometries have been applied to avoid beam directions intersecting with a dental material, or treatment volumes have been modified to exclude dental materials. If such avoidance strategies are not feasible in certain cases, particle therapy may even be contraindicated.^19^, ^20^ Thus, the restrictions posed on treatment planning by dental materials could compromise treatment plan quality^20^ and therapy outcome. Size, shape, material composition, relative electron density (RED), and effective atomic number (EAN) of dental materials are usually not characterized at the time of radiotherapy planning.^5^ On the other hand, with an improved physical characterization of common dental materials and understanding of their dosimetric impact, it may become possible to use more conventional treatment planning strategies for accurately contoured dental materials.^19^ This procedure may allow the optimizer to compensate for the materials themselves rather than simply avoiding dental materials.^20^

Previous investigations have employed various approaches to manage non‐tissue implant materials. Imaging approaches, such as metal artifact reduction (MAR) methods, have been used to reduce CT imaging artifacts; other approaches have been applied to optimize treatment planning procedures, as, for example, when the required avoidance margin is determined in order to assure that the implant does not affect the dose distribution.^6^, ^21^, ^22^, ^23^, ^24^, ^25^, ^26^, ^27^, ^28^, ^29^, ^30^, ^31^ Evaluations have mostly been limited to photon therapy, even though some studies addressed dosimetric uncertainties for particle therapy.^6^, ^20^, ^24^, ^29^, ^31^ Recently, Hu et al. investigated common dental materials in terms of relative stopping power and 3D dose perturbation.^20^ Thereby, all investigated dental materials substantially perturbed the dosimetry of pristine proton spots with respect to relative stopping power and spatial dose distribution.^20^ Monte Carlo simulations and TPS dose calculations demonstrated good agreement with measurements, suggesting potential for proton treatments through dental materials given further investigation.^20^ Because dental materials are a common scenario in particle therapy, further research is needed to improve treatment planning for patients with dental materials. In this context, DECT‐based SPR prediction might be beneficial, but has not been sufficiently investigated hitherto.

This study examines the impact of using DECT imaging for particle therapy treatment planning in patients with dental materials regarding three aspects. (i) First, radiological material parameters of commonly used dental implant and restoration materials are determined with two different DECT techniques, using image‐based (SACT) and projection‐based (DLCT) methods. (ii) Second, through comparison of SECT‐ and DECT‐based SPR predictions in phantoms with measured reference data, compensation of dental materials in particle therapy planning and feasibility of ion beam delivery are investigated. (iii) Third, DECT‐based particle therapy treatment planning for head and neck cancer patients for one exemplary dental material is evaluated in a head phantom.

## 2 MATERIALS AND METHODS

### 2.1 Dental materials

**FIGURE 1:**
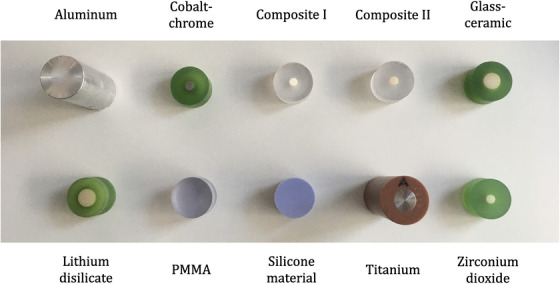
Cylindrical samples of investigated dental materials: aluminum, cobalt‐chrome, composite I, composite II, glass‐ceramic, lithium disilicate, polymethyl methacrylate (PMMA), silicone material, titanium, and zirconium dioxide. Some samples were fabricated as an inner core surrounded by PMMA (in green color or transparent).

Dental implant and restoration materials were selected in consultation with the institutional department of prosthodontics. Commonly used materials for fixed dental prostheses (cobalt‐chrome, lithium disilicate, zirconium dioxide), core buildups/fillings (composite I), direct restorations/fillings (composite II), veneers/inlays/partial crowns (glass‐ceramic), and dental implants (titanium, zirconium dioxide) were chosen for investigation. All these materials comprised elements of a high atomic number *Z*: cobalt‐chrome (*Z_Co_* = 27, *Z_Cr_* = 24), composite I (*Z_Ba_* = 56), composite II (*Z_Yb_* = 70), glass‐ceramic (*Z_Y_* = 39), lithium disilicate (*Z_Zn_* = 30, *Z_Zr_* = 40), titanium (*Z_Ti_* = 22), zirconium dioxide (*Z_Zr_* = 40). Furthermore, we analyzed the single components of individualized 3D‐printed tissue retraction devices (TRDs) used to protect healthy tissue from irradiation in head and neck radiotherapy.^32^, ^33^ TRDs consist of a fixation part (made of silicone material) and a tongue retraction part (made of polymethyl methacrylate [PMMA]). Samples were fabricated into cylinders with a diameter of 2.8 cm and a length of 1−2 cm (depending on the fabrication abilities) (Figure 1). Thereby, samples of cobalt‐chrome, composites, glass‐ceramic, lithium disilicate, and zirconium dioxide were fabricated as an inner core with a diameter of 6−13 mm surrounded by PMMA due to manufacturing capabilities or the thickness of the materials. Additionally, a titanium insert from the Gammex 467 phantom (Gammex Electron Density CT Phantom 467, Gammex‐RMI, Middleton, WI, USA) as well as an aluminum insert were used. Even though pure aluminum is not a dental material, it was chosen for investigation since it is a component of aluminum oxide ceramics, which are obsolete now but were previously used for dental restorations. Additionally, a pure metal (*Z_Al_* = 13) might be of interest for other applications. For simplicity, aluminum is listed as dental material in this study. Details about the investigated materials can be found in Supplementary Table S1.

### 2.2 DECT imaging techniques and CT image acquisition and reconstruction

Two different DECT imaging techniques available at the Heidelberg University Hospital were applied: a SACT scanner (SOMATOM Confidence, Siemens Healthcare GmbH, Erlangen, Germany) and a diagnostic DLCT scanner (Spectral CT 7500, Philips Healthcare, Best, The Netherlands).

SACT acquires the entire volume sequentially at two different tube potentials to generate dual‐energy data with a single‐source CT scanner. Hence, SACT image data can be acquired on any existing CT scanner; however, dedicated dual‐energy acquisition modes are required. Due to the two sequential image acquisitions at two different tube potentials, SACT is able to provide very high spectral separation, but has a low temporal coherence (large offset). Material decomposition needs to be image‐based.

DLCT uses a single x‐ray tube paired with a double‐layer detector that simultaneously detects projection‐aligned high‐ and low‐energy x‐ray data. Each layer of the detector has a maximum sensitivity for different energy spectra. Thus, DLCT implicitly acquires dual‐energy data without the need for a special mode, and allows for the pro‐ and retrospective generation of results with a perfect temporal and spatial alignment over the full field‐of‐view. However, medium spectral separation is expected to be achieved and cross‐scatter between detector layers may occur. Projection‐based material decomposition with implicit noise‐reduction exploiting anti‐correlated noise in both detector layers can be performed with DLCT imaging.

The image acquisition and reconstruction protocols for the two DECT techniques are based on current state‐of‐the‐art clinical head protocols for treatment planning at the Heidelberg Ion Beam Therapy Center (HIT, Germany) (Supplementary Table S2). For SACT systems, dedicated DECT image acquisition and reconstruction protocols are necessary. For DLCT systems, the same protocol as for conventional imaging can be used to simultaneously obtain conventional and spectral image data. Additionally, for each SACT image acquisition, the Siemens iterative metal artifact reduction algorithm (iMAR) (Siemens Healthcare GmbH, Erlangen, Germany), and for each DLCT image acquisition, the Philips orthopedic metal artifact reduction algorithm (O‐MAR) (Philips Healthcare, Best, The Netherlands) were applied,^29^, ^34^ since these MAR algorithms are directly implemented on the used clinical CT scanners.

### 2.3 Investigation of radiological material parameters of dental materials

Image data of dental materials were acquired in a custom one‐bore cylindrical phantom of 46.0 cm height and 8.0 cm radius made of PMMA to mimic beam hardening that occurs in a typical situation in the head. To ensure that no signal from one material interfered with the measurement signal from an adjacent material, a distance of 8 cm (detector width of the Spectral CT 7500) or 2 cm (detector width of the SOMATOM Confidence CT) was left between the individual dental materials in the phantom. The radiological material parameters CT number (CTN), RED, and EAN data provided by SACT and DLCT image data were quantified for initial characterization of the dental materials. RED and EAN datasets were obtained from SACT acquisitions using the module syngo.CT DE Rho/Z in the syngo.via environment (Siemens Healthcare GmbH, Erlangen, Germany) and from DLCT acquisitions using Philips spectral software (Philips Healthcare, Best, The Netherlands). Circular regions‐of‐interest (ROIs) with a size of ∼70% of the inserts’ cross‐sectional diameters were placed in axial CT slices of each dental material. By using this method, possible artifacts that may arise due to gradient effects to the surrounding PMMA near the material–phantom boundary were avoided. For similar reasons, CT slices at both ends of the investigated materials were also excluded. The mean and standard deviation of the extracted values over all slices from each dental material were calculated.

Furthermore, the DEEDZ‐MD method proposed by Saito^35^ was used to calculate the mass density (MD, *ρ*) from DECT‐based RED (*ρ_e_*) and EAN (*Z_eff_*) values, with *Z_eff,w_* being the EAN of water:

(1) $$\rho =\rho_{e}+\rho_{e}\sum_{n\;=\;0}^{2}e_{n}{\left\{{\left(\frac{Z_{eff}}{Z_{eff,w}}\right)}^{m}-1\right\}}^{n}$$

According to Saito and Sagara,^36^ the value of m was set to 3.3, and the same human tissue‐specific parameters (*e_n_*) as obtained by Saito^35^ were used. The DEEDZ‐MD method was first experimentally validated for both DECT techniques using tissue‐equivalent inserts (Gammex Electron Density CT Phantom 467, Gammex‐RMI, Middleton, WI, USA) and compared with MD data provided by the vendor (relative mean deviation of −1.2% [SACT] and −1.4% [DLCT]) before being applied to dental materials.

Besides, the severity of artifacts was qualitatively evaluated on a four‐point scale^37^: 1, no; 2, mild; 3, moderate; 4, severe artifacts.

### 2.4 Measurement and prediction of SPR values for dental materials

#### 2.4.1 Calculation of predicted SPR values based on quantitative DECT data

For DLCT imaging, RED and EAN data of the spectral results were used to calculate SPR values via the Bethe equation using an in‐house program (denoted as DE‐RhoZ‐DLCT).^18^ The mean excitation energy (I‐value) was estimated from a piecewise linear fit to EAN using the method proposed by Yang et al.^38^ The mean excitation energy of water was set to 78.73 eV, consistent with the values proposed by Bär et al.^39^ and the ICRU Report 90.^40^ Following the recommendation of Inaniwa and Kanematsu,^41^ a fixed particle kinetic energy of 100 MeV per nucleon was assumed, because the energy dependence of SPR prediction in the therapeutic range is minimal.^42^

For SACT imaging, a DirectSPR implementation (Siemens Healthcare GmbH, Erlangen, Germany) in the syngo.via image‐reconstruction software was employed (DE‐DirectSPR‐SACT).^43^ In addition, RED and EAN data were obtained from the module syngo.CT DE Rho/Z were used to calculate SPR values via the same procedure as explained for DLCT imaging (referred to as DE‐RhoZ‐SACT).

**FIGURE 2:**
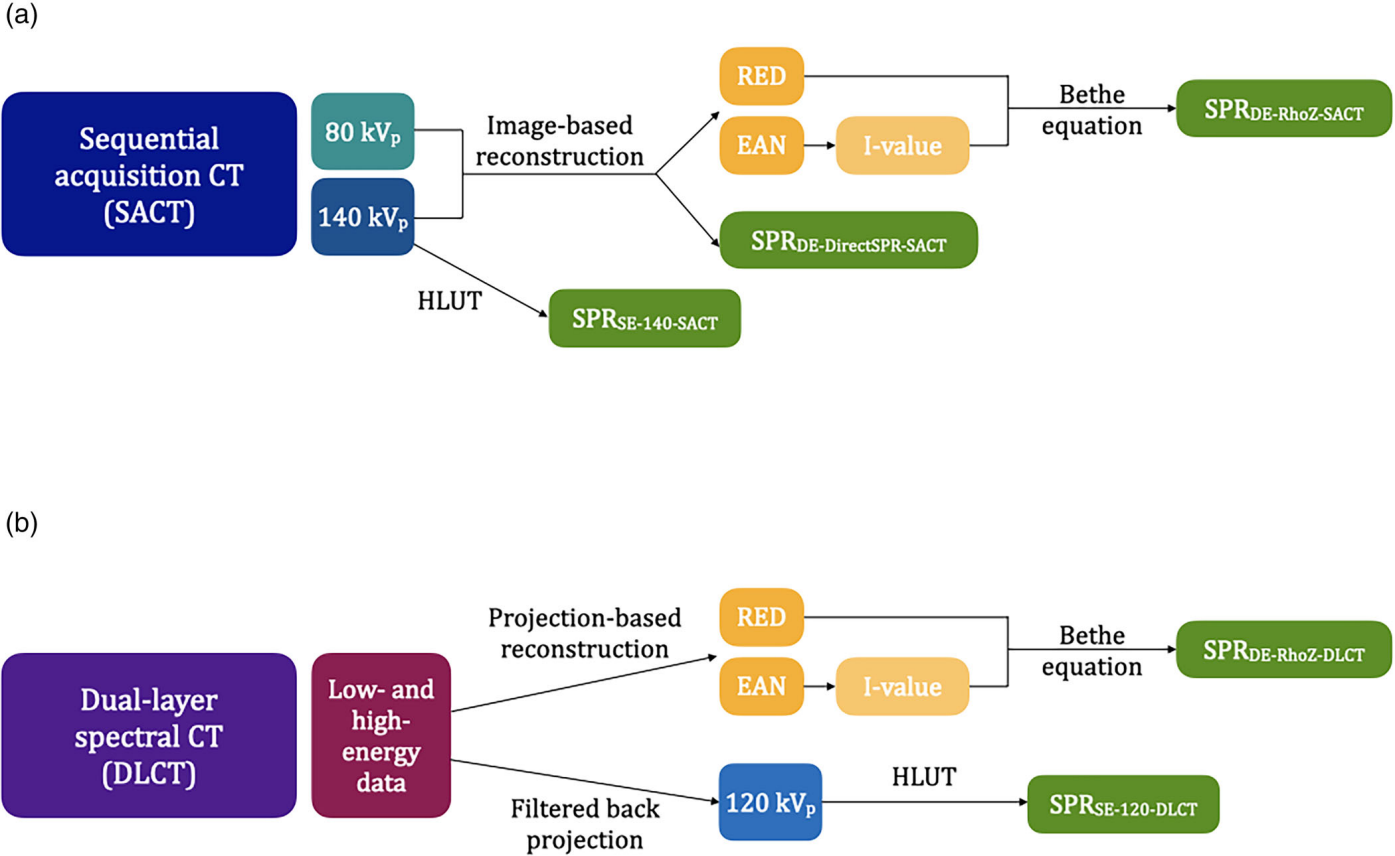
Practical implementation of (a) sequential acquisition CT (SACT) and (b) dual‐layer spectral CT (DLCT) for single‐energy CT (SECT)‐ and dual‐energy CT (DECT)‐based stopping power ratio (SPR) prediction using relative electron density (RED), effective atomic number (EAN), and mean excitation energy (I‐value), or a Hounsfield look‐up table (HLUT). SE‐140‐SACT, SECT‐based SPR prediction with SACT at 140 kV_p_; DE‐DirectSPR‐SACT, DECT‐based SPR prediction with SACT using a DirectSPR implementation; DE‐RhoZ‐SACT, DECT‐based SPR prediction with SACT using the RhoZ‐method; SE‐120‐DLCT, SECT‐based SPR prediction with DLCT at 120 kV_p_; DE‐RhoZ‐DLCT, DECT‐based SPR prediction with DLCT using the RhoZ‐method.

Validation of SPR prediction for both DECT techniques (SACT and DLCT) and SPR prediction methods (DE‐DirectSPR and DE‐RhoZ) was performed using tissue‐equivalent inserts (Gammex Electron Density CT Phantom 467, Gammex‐RMI, Middleton, WI, USA) in a cylindrical PMMA phantom before applying DECT‐based SPR prediction to dental materials. Relative mean deviation compared to measured SPR values was below 0.7% for DE‐DirectSPR‐SACT, DE‐RhoZ‐SACT, and DE‐RhoZ‐DLCT. Figure 2 illustrates the practical implementation of SECT and DECT approaches used in this study.

#### 2.4.2 Calculation of predicted SPR values based on conventional SECT image data

For the protocols of each of the two DECT techniques, a HLUT converting CTN to SPR values was generated. The clinically applied procedure using tissue‐equivalent inserts (Gammex Electron Density CT Phantom 467, Gammex‐RMI, Middleton, WI, USA) was followed. For DLCT, the acquired 120 kV_p_ SECT image data was used (SE‐120‐DLCT), whereas for SACT, the HLUT was based on the 140 kV_p_ SECT image data (SE‐140‐SACT). The HLUTs were created based on the two‐parameter stoichiometric parametrization,^44^, ^45^ following the current clinical protocol at HIT.^46^

#### 2.4.3 Measurement of SPR values

SPR values of all dental materials were determined experimentally at HIT by measuring, from each material of interest, the shift of a Bragg peak in a water absorber (Peakfinder Water Column, PTW‐Freiburg, Freiburg, Germany) using carbon ions at 250.1 MeV/u. Carbon ions were used for the SPR measurements due to their sharper Bragg peak, reduced lateral scattering, and less range straggling compared to protons.^17^ The measured SPR was calculated as:

(2) $$\;SPR_{meas}=\frac{P_{w}-P_{m}}{d_{m}}\;$$

Here, *P_w_* denotes the mean of the depths in the water absorber corresponding to the fitted dose maximum, dose maximum, 90% distal dose, and 80% distal dose without a dental material sample present. *P_m_* is the mean of the four depths with a dental material sample m intersecting the beam, and d is the thickness of the sample.

#### 2.4.4 Assessment of DECT‐ and SECT‐based SPR predictions

Relative residuals were calculated to quantify the deviation between measured reference (*SPR_meas_*) and SECT‐ or DECT‐predicted SPR values (*SPR_CT_*):

(3) $$relative\;residual=\frac{SPR_{CT}-SPR_{meas}}{SPR_{meas}}\;\cdot 100\%$$

### 2.5 Assessment of treatment planning using DECT‐based SPR prediction for ion beams with an anthropomorphic head phantom containing dental materials

**FIGURE 3:**
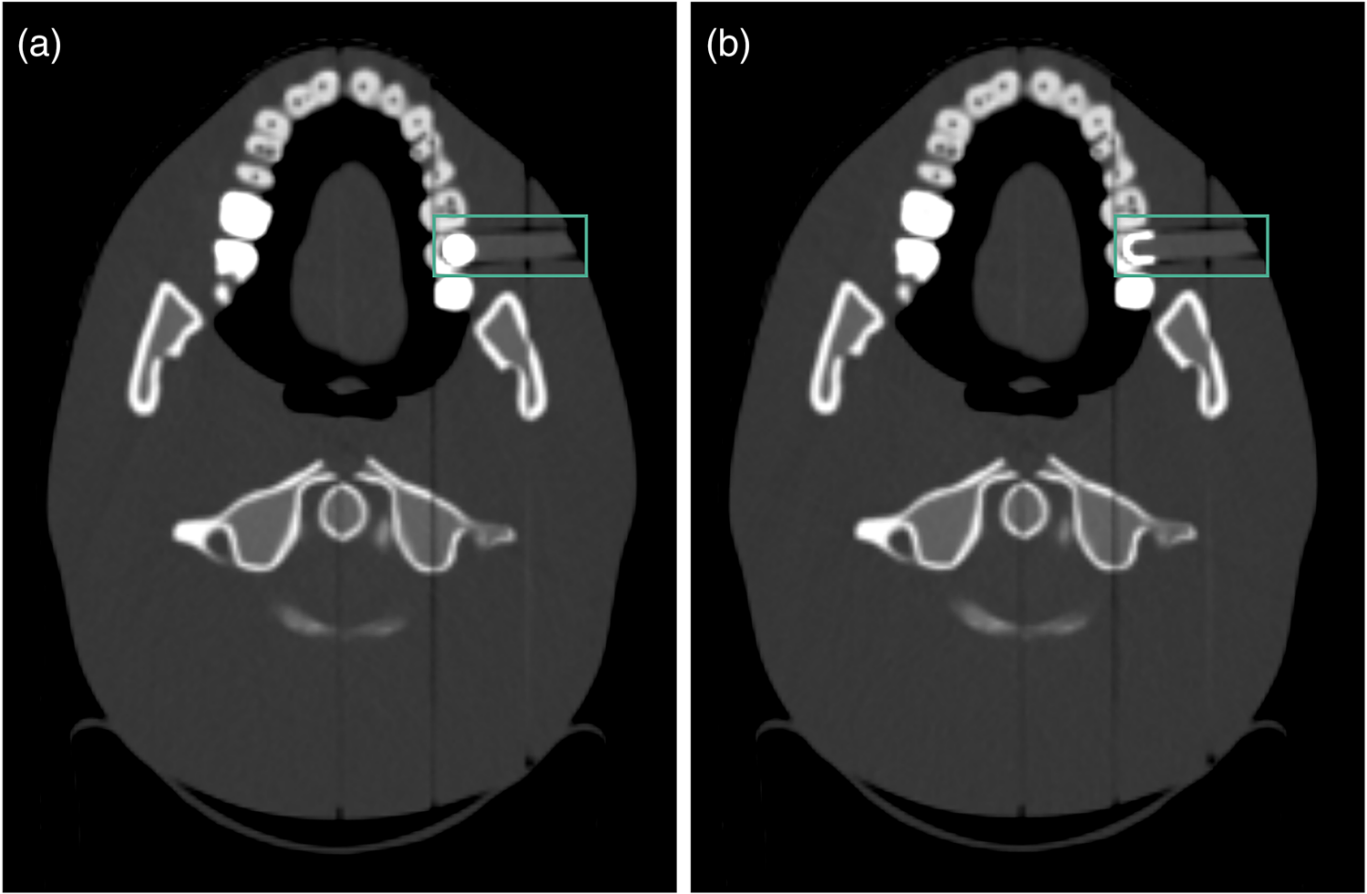
CT images of the anthropomorphic head phantom with a removable tooth in two different configurations: (a) spherical dental material and (b) dental crown.

To assess particle therapy treatment planning accuracy for patients with dental materials in clinical‐like conditions, range prediction and dosimetric impact were evaluated using a tissue‐equivalent anthropomorphic head phantom (Proton Therapy Dosimetry Head, Model 731‐HN, Computerized Imaging Reference Systems, Inc. (CIRS), Norfolk, VA, USA). The model contains two removable pins that allow segments of molar tooth roots to be exchanged. The segments are made of either tungsten or tissue‐equivalent material. For this study, one of the teeth, located beneath different tissue‐equivalent layers, was replaced with a dental restoration made of lithium disilicate. Lithium disilicate is a common dental material from Section 2.1 which was selected for further investigation in clinical‐like conditions (Figure 3). Two different custom‐made types of one of the replaceable teeth were fabricated: (a) spherical dental material with a diameter of 7 mm on a PMMA basis and (b) dental crown with a thickness of 1 mm on a PMMA basis. The head phantom was immobilized with a head cushion and an individualized thermoplastic mask, and the part of the mask surrounding the tooth was cut off.

Helium ions were chosen for the investigation of range and dose differences due to their intermediate physical properties between proton and carbon ion beams.^47^ Helium ions have a sharper Bragg peak, less multiple Coulomb scattering, and reduced range straggling in comparison with protons, but a smaller fragmentation tail than carbon ions. Instead of optimizing a treatment plan for a specific target volume, one iso‐energy layer at 92.75 MeV/u was created with the RayStation TPS version 11B (RaySearch Laboratories AB, Stockholm, Sweden) using a pencil beam dose engine with a dose grid of 1 mm and spot spacing of 2 mm.

In all CT datasets, the PMMA basis and volume behind the dental material were manually delineated and overridden with the measured SPR value of PMMA (cf. Section 2.4.3). In an additional SE‐140‐SACT dataset, the dental material volume was delineated and overridden with the experimentally determined SPR value as well. Because the beam only passes through the experimentally determined overridden materials without passing any additional tissue‐equivalent material, this material‐specific dataset was then assumed to be the (measured) reference dataset to be compared with other CT datasets. Consequently, range differences that might occur between different CT datasets are only due to the dental restoration itself.

The iso‐energy layer was initially calculated on the reference dataset. Following plan optimization, forward dose calculations were performed on five additional (image) datasets using the (A) SE‐140‐SACT (without material override of the dental material), (B) DE‐DirectSPR‐SACT, (C) DE‐RhoZ‐SACT, (D) SE‐120‐DLCT, and (E) DE‐RhoZ‐DLCT approach.

SPR predictions from all CT datasets of the head phantom were analyzed with ROIs in the dental material (Figure 3a) as well as in the tooth dentin and tooth enamel. The dental material was compared to measured reference values acquired in Section 2.4.3 and the tooth dentin and tooth enamel were compared to measured values of 1.501 ± 0.003 and 1.763 ± 0.003 determined by Wohlfahrt et al.^16^ Physical dose distributions calculated with SECT and DECT were then compared in terms of their range prediction to the (measured) reference dataset, respectively. Since range differences in the head phantom were analyzed and quantified in previous studies for beams unobstructed by dental material,^16^, ^17^, ^18^ this study focused on evaluating range differences arising from the dental material. Differences in range prediction between (image) datasets and the reference dataset were analyzed with line‐dose profiles in beam direction and quantified by absolute range shifts at the distal range at 80% (R_80_) of prescribed dose (ΔR_80_ = R_80,Reference_ − R_80,CT_).

## 3 RESULTS

### 3.1 Determination of radiological material parameters of dental materials

The radiological parameters CTN, RED, EAN, MD, and artifact categories of the dental materials for the two DECT acquisition techniques are presented in Table 1. Dental materials containing an element of a high atomic number (cobalt‐chrome, composites I and II, glass‐ceramic, lithium disilicate, titanium, and zirconium dioxide) saturated or nearly saturated CTN using SACT or DLCT and caused streak artifacts. RED, EAN, and MD values of TRD materials were similar for SACT and DLCT (relative deviation was <0.8% for RED, <3.0% for EAN, <1.5% for MD). However, RED, EAN, and thus also MD values differed for dental implant and restoration materials between SACT and DLCT because of their different (image‐ and projection‐based) calculation methods.

**TABLE 1:** Measured mean values and standard deviation of CT number (CTN), relative electron density (RED), effective atomic number (EAN), mass density (MD), and artifact category of dental materials for sequential acquisition CT (SACT) and dual‐layer spectral CT (DLCT)

Material	CTN_SACT_ (HU)	CTN_DLCT_ (HU)	RED_SACT_	RED_DLCT_	EAN_SACT_	EAN_DLCT_	MD_SACT_ (g/cm^3^)	MD_DLCT_ (g/cm^3^)	Artifact category
Aluminum	2047 ± 4	2279 ± 8	2.423 ± 0.002	2.331 ± 0.012	12.88 ± 0.05	13.00 ± 0.01	2.578 ± 0.002	2.485 ± 0.005	Mild
Cobalt‐chrome	3030 ± 8	>3071	3.980 ± 0.006	4.049 ± 0.001	7.31 ± 0.05	16.00 ± 0.01	4.001 ± 0.005	4.516 ± 0.001	Severe
Composite I	3037 ± 4	>3071	3.898 ± 0.006	2.072 ± 0.088	7.79 ± 0.02	19.71 ± 0.49	3.929 ± 0.010	2.376 ± 0.088	Severe
Composite II	3026 ± 3	>3071	3.967 ± 0.007	4.049 ± 0.001	7.41 ± 0.03	16.00 ± 0.01	3.990 ± 0.006	4.516 ± 0.001	Severe
Glass‐ceramic	2648 ± 6	>3071	3.282 ± 0.007	2.342 ± 0.003	10.38 ± 0.02	16.00 ± 0.01	3.382 ± 0.005	2.612 ± 0.003	Mild
Lithium disilicate	3069 ± 1	>3071	3.991 ± 0.022	2.583 ± 0.004	7.78 ± 0.11	17.00 ± 0.01	4.023 ± 0.024	2.919 ± 0.004	Mild
PMMA	127 ± 1	125 ± 2	1.154 ± 0.002	1.152 ± 0.001	6.38 ± 0.06	6.50 ± 0.55	1.155 ± 0.003	1.153 ± 0.001	No
Silicone material	431 ± 1	509 ± 3	1.268 ± 0.006	1.259 ± 0.002	10.68 ± 0.06	11.00 ± 0.01	1.311 ± 0.006	1.307 ± 0.001	No
Titanium	3069 ± 1	>3071	3.976 ± 0.013	4.026 ± 0.014	8.09 ± 0.09	16.00 ± 0.01	4.016 ± 0.013	4.490 ± 0.014	Moderate
Zirconium dioxide	3023 ± 7	>3071	4.000 ± 0.005	4.049 ± 0.001	7.18 ± 0.06	16.00 ± 0.01	4.018 ± 0.005	4.516 ± 0.001	Severe

*Note*: The CTN for SACT were determined on 140 kV_p_ image datasets. Since no extended HU scale was used, >3071 HU means that the CTN scale was saturated.

### 3.2 Measurement and prediction of SPR values for dental materials

**FIGURE 4:**
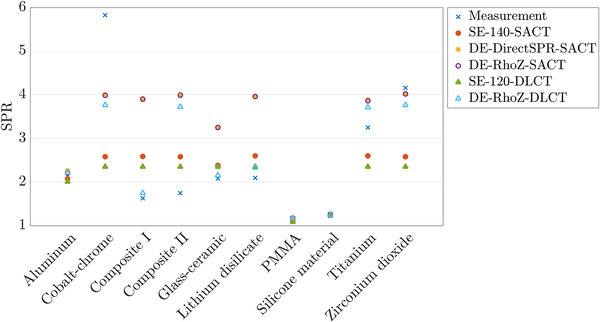
Stopping power ratio (SPR) values of the investigated dental materials predicted with sequential acquisition CT (SACT) and dual‐layer spectral CT (DLCT). SE‐140‐SACT, SECT‐based SPR prediction with SACT at 140 kV_p_; DE‐DirectSPR‐SACT, DECT‐based SPR prediction with SACT using a DirectSPR implementation; DE‐RhoZ‐SACT, DECT‐based SPR prediction with SACT using the RhoZ‐method; SE‐120‐DLCT, SECT‐based SPR prediction with DLCT at 120 kV_p_; DE‐RhoZ‐DLCT, DECT‐based SPR prediction with DLCT using the RhoZ‐method. Note that the markers of DE‐DirectSPR‐SACT are hidden by overlaying markers of DE‐RhoZ‐SACT.

Figure 4 presents SPR values of the investigated dental materials together with the respective CT acquisition technique (SACT and DLCT) and calculation method as well as measured SPR values, which are additionally listed in Supplementary Table S3. Comparison of SECT‐ and DECT‐predicted SPR values are given in Table 2. For dental implant and restoration materials, SPR prediction accuracy using DECT techniques was overall closer to measured values than SECT, although DECT methods showed limitations for materials containing elements of a high atomic number. For TRD materials, DECT‐based SPR prediction accuracy compared to measured SPR was <0.7% for PMMA and <2.3% for silicone material.

**TABLE 2:** Relative residuals of dual‐energy CT (DECT)‐ and single‐energy CT (SECT)‐predicted stopping power ratio (SPR) values of dental materials from measured SPR values in % using sequential acquisition CT (SACT) and dual‐layer spectral CT (DLCT)

	SACT—Relative residuals			DLCT—Relative residuals	
Material	SPR_SE‐140_	SPR_DE‐DirectSPR_	SPR_DE‐RhoZ_	SPR_SE‐120_	SPR_DE‐RhoZ_
Aluminum	−3.08	5.98	2.93	−6.29	3.83
Cobalt‐chrome	−55.68	−31.36	−31.56	−59.69	−35.43
Composite I	58.88	138.78	139.56	44.25	7.52
Composite II	47.88	127.68	128.78	34.58	113.38
Glass‐ceramic	14.67	55.96	56.38	12.89	3.65
Lithium disilicate	24.39	89.79	89.44	12.24	11.53
PMMA	−6.57	0.49	0.68	−6.93	0.61
Silicone material	1.06	2.03	2.28	1.09	1.35
Titanium	−19.92	19.40	18.94	−27.74	14.17
Zirconium dioxide	−38.05	−3.31	−3.40	−43.58	−9.59

Abbreviations: SE‐140‐SACT, SECT‐based SPR prediction with SACT at 140 kV_p_; DE‐DirectSPR‐SACT, DECT‐based SPR prediction with SACT using a DirectSPR implementation; DE‐RhoZ‐SACT, DECT‐based SPR prediction with SACT using the RhoZ‐method; SE‐120‐DLCT, SECT‐based SPR prediction with DLCT at 120 kV_p_; DE‐RhoZ‐DLCT, DECT‐based SPR prediction with DLCT using the RhoZ‐method.

For SACT, the difference in SPR prediction between DE‐DirectSPR‐SACT and DE‐RhoZ‐SACT methods was minimal. Since the DE‐RhoZ‐SACT and DE‐DirectSPR‐SACT algorithms rely on two image datasets acquired at 80 kV_p_ and 140 kV_p_, it becomes difficult to achieve reliable quantitative DECT data if CTN of one or both of the 80 kV_p_ and 140 kV_p_ acquisitions are nearly or fully saturated (CTN ≥ 3071 HU) (cf. Table 1). This might cause the larger variations in SPR prediction compared to measured SPR values for cobalt‐chrome, composites I and II, glass‐ceramic, lithium disilicate, and titanium. Using the MAR algorithm iMAR showed no considerable difference in DECT‐based SPR prediction within dental materials (deviation <0.2%).

For DLCT, DECT‐based SPR predictions showed better agreement compared to measured SPR values than SECT‐based SPR predictions for all dental materials (excluding composite II). DE‐RhoZ‐DLCT‐predicted SPR for composite II showed a larger deviation than the other dental materials, which could result from a trace of the element ytterbium of a high atomic number. DECT‐based SPR prediction accuracy for dental materials was similar with and without the MAR algorithm O‐MAR (deviation <0.2%).

### 3.3 Assessment of treatment planning using DECT‐based SPR prediction for ion beams with an anthropomorphic head phantom containing dental materials

**FIGURE 5:**
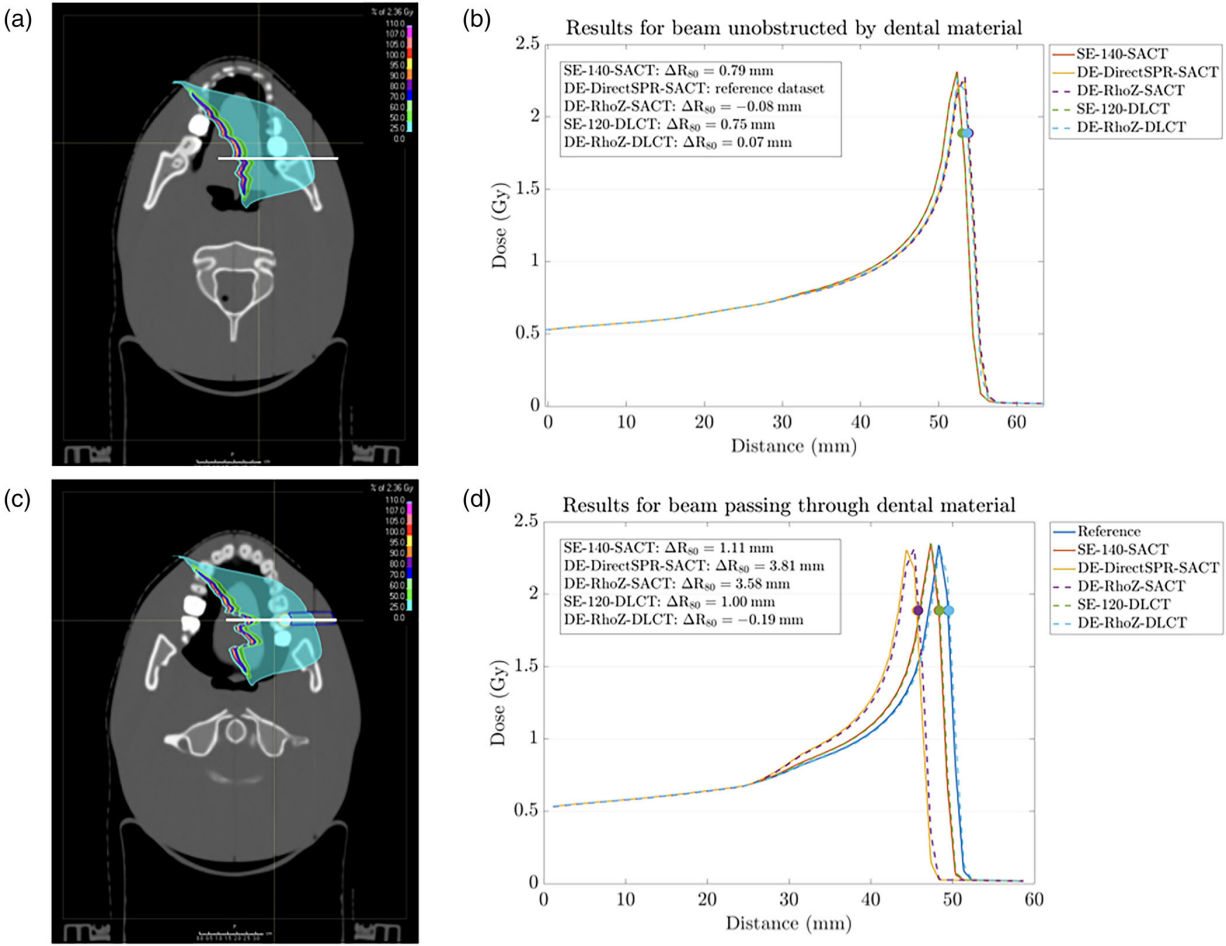
For two different axial CT slices, dose distribution for helium ions (a,c) and two representative line‐dose profiles calculated on sequential acquisition CT (SACT) and dual‐layer spectral CT (DLCT) to quantify deviations in range prediction (b,d) for a beam unobstructed by (a,b) or passing through (c,d) dental material. The placement of the line‐dose profiles in (b,d) is illustrated in (a,c). The illustrated depth‐dose curves indicate in beam's eye view absolute range (R) differences at R_80_ (marked with circles) between DE‐DirectSPR‐SACT and the other stopping power ratio (SPR) prediction methods in (b) and between the reference dataset and the different SPR prediction methods in (d). SE‐140‐SACT, SECT‐based SPR prediction with SACT at 140 kV_p_; DE‐DirectSPR‐SACT, DECT‐based SPR prediction with SACT using a DirectSPR implementation; DE‐RhoZ‐SACT, DECT‐based SPR prediction with SACT using the RhoZ‐method; SE‐120‐DLCT, SECT‐based SPR prediction with DLCT at 120 kV_p_; DE‐RhoZ‐DLCT, DECT‐based SPR prediction with DLCT using the RhoZ‐method.

In the first part, range prediction differences for beams unobstructed by dental material were analyzed. As an initial validation, SECT‐based predictions from both CT acquisition techniques (SACT and DLCT) were compared and showed similar range predictions with maximum differences <0.2 mm at R_80_ for beams unobstructed by dental material (Figure 5). These minor differences could be due to image registration and slightly different HLUTs of the two CT imaging protocols. Next, DE‐DirectSPR‐SACT predictions were investigated and confirmed range differences compared to SE‐140‐SACT‐based SPR predictions. Since Wohlfahrt et al.^16^ found that DirectSPR methods with a Siemens SACT scanner performed better than SECT‐based SPR prediction methods in the CIRS anthropomorphic head phantom using validated ground truth SPR data, we also assumed that DE‐DirectSPR‐SACT predicts SPR values closer to the ground truth than SECT. The assumption that DE‐DirectSPR‐SACT outperforms SECT in the head phantom was confirmed by analyzing SPR values in the tooth dentin and tooth enamel (Table 3). Analyzed line‐dose profiles revealed that both investigated DE‐SACT approaches used in this study (DE‐DirectSPR and DE‐RhoZ) predicted similar ranges with small differences of <0.4 mm at R_80_. As a following step, DE‐DLCT range predictions were analyzed and found to be similar to DE‐SACT for beams unobstructed by dental material with range differences between DE‐SACT and DE‐DLCT of <0.5 mm. Range differences might emerge from the two different DECT techniques (image‐ and projection‐based methods), although small differences could also result from the image registration and image resolution. No systematic shift in one direction was observed. Thus, SECT and DECT predictions of the two CT acquisition techniques each showed to predict similar ranges in the head phantom (Figure 5). A previous study showed that DE‐RhoZ‐DLCT‐predicted dose distributions revealed higher 3D gamma passing rates compared to SE‐120‐DLCT‐predicted dose distributions for a helium ion therapy plan using the anthropomorphic head phantom.^18^ Based on this, DE‐RhoZ‐DLCT SPR calculations were considered to be closer to the reference values―in alignment with the results that DE‐DirectSPR‐SACT outperforms SE‐140‐SACT.

**TABLE 3:** Relative residuals of dual‐energy CT (DECT)‐ and single‐energy CT (SECT)‐predicted stopping power ratio (SPR) values from measured SPR values in % using sequential acquisition CT (SACT) and dual‐layer spectral CT (DLCT) in the anthropomorphic head phantom

	SACT—Relative residuals			DLCT—Relative residuals	
Material	SPR_SE‐140_	SPR_DE‐DirectSPR_	SPR_DE‐RhoZ_	SPR_SE‐120_	SPR_DE‐RhoZ_
Tooth dentin	1.53	−0.25	−0.33	1.53	−0.29
Tooth enamel	10.94	−0.58	−0.57	10.89	−0.97
Lithium disilicate restoration	10.91	35.04	34.27	8.42	0.22

*Note*: SPR values were determined in regions‐of‐interest (ROIs) in the tooth dentin, tooth enamel, and lithium disilicate restoration.

Abbreviations: SE‐140‐SACT, SECT‐based SPR prediction with SACT at 140 kV_p_; DE‐DirectSPR‐SACT, DECT‐based SPR prediction with SACT using a DirectSPR implementation; DE‐RhoZ‐SACT, DECT‐based SPR prediction with SACT using the RhoZ‐method; SE‐120‐DLCT, SECT‐based SPR prediction with DLCT at 120 kV_p_; DE‐RhoZ‐DLCT, DECT‐based SPR prediction with DLCT using the RhoZ‐method.

In the second part, range prediction differences for ion beams passing through the dental material (Figure 3a) were analyzed. The lithium disilicate restoration had a smaller (more patient‐realistic) diameter than the sample used in Section 3.2, which resulted in different SPR prediction accuracies (Tables 2 and 3). DE‐SACT (DE‐DirectSPR‐SACT and DE‐RhoZ‐SACT) overestimated the SPR value of the lithium disilicate restoration (Table 3), which was already seen in Figure 4 and Table 2. This might result from partially saturated CTN in the 80 kV_p_ image datasets of the dental material. For this specific material, SECT‐based SPR prediction was closer to the measured SPR value. Therefore, in its current status, DE‐SACT‐based SPR prediction for a lithium disilicate restoration may not be accurate enough for dose prediction in particle therapy. However, this also depends on the material, size, and shape of the dental restoration and how much of the dental material is in the beam direction. The use of a lithium disilicate dental crown with a thickness of 1 mm on a PMMA basis (Figure 3b) resulted in an underestimation of the SPR value with DE‐SACT due to beam hardening effects, but may lead to smaller overall range prediction errors due to the smaller diameter of the dental material. Projection‐based SPR prediction for the lithium disilicate restoration using DE‐RhoZ‐DLCT was closer to the measured SPR than SE‐120‐DLCT (Table 3) and resulted in a remaining range deviation of 0.2 mm (SE‐120‐DLCT: 1.0 mm) at R_80_ compared to the reference dataset (Figure 5).

## 4 DISCUSSION

### 4.1 Key findings

In this study, we investigated the use of DECT‐based particle therapy treatment planning for patients with dental implant and restoration materials, which is a common scenario in head and neck particle therapy. The reduction of dental material‐induced effects is challenging, and is still an active research area because dental materials may negatively impact particle therapy treatment planning. Dental implant or restoration materials are composed of various materials such as cobalt‐chrome, composites, glass‐ceramic, lithium disilicate, titanium, and zirconium dioxide (Supplementary Table S1). In this study, DECT image acquisitions were performed with two different DECT techniques using SACT and DLCT scanners, each showing strengths and limitations for application in particle therapy. A comparison of different DECT acquisition techniques with a focus on particle therapy treatment planning has been described in previous works^8^, ^9^, ^10^, ^18^ and is summarized in Section 2.2.

This study is the first to comprehensively investigate a full spectrum of common dental materials. Radiological material parameters CTN, RED, EAN, MD, and artifact categories of ten common dental materials currently applied in prosthetic and restorative dentistry and in radiotherapy‐related tissue retraction were determined. CTN for most of the materials were larger than 3000 HU and some even saturated the CTN scale, causing mild to severe artifacts (Table 1).

Measured SPR values ranged from 1.169 (PMMA) to 5.823 (cobalt‐chrome) (Figure 4 and Supplementary Table S3). For TRD materials, DECT can improve SPR prediction accuracy for particle therapy planning, resulting in remaining discrepancies of <0.7% for PMMA and <2.3% for silicone material (Table 2). In cases where TRDs lie in the beam path, dose delivered in tissue around TRDs can be more accurately predicted by using DECT.

For dental materials, DECT showed overall SPR predictions closer to measured SPR than SECT (Table 2). For cobalt‐chrome (metal alloy), titanium (pure metal), and zirconium dioxide, which are materials made of elements of a high atomic number, both DE‐SACT‐ and DE‐DLCT‐based SPR predictions seemed to reach a plateau at values around 3.9 (DE‐SACT) and 3.7 (DE‐DLCT) (Supplementary Table S3). These values seemed to be the current upper limit of the investigated DECT reconstruction methods. Furthermore, these elements do not only feature higher atomic numbers, but also high mass and electron densities relative to the natural elemental composition of the human body. The measured values displayed in Figure 4 indicate strong influence of (electron) density for these materials offering a potential explanation for the deviations between measurements and CT imaging‐based SPR prediction. Thus, it remained challenging to accurately predict SPR values of these materials exhibiting high SPR values. Yet, the use of DECT improved SPR prediction for cobalt‐chrome, titanium, and zirconium dioxide compared to SECT. The two HLUTs applied have maximum SPR values of 2.602 (SE‐140‐SACT) and 2.347 (SE‐120‐DLCT), which led to an SPR underestimation of up to 60% for these dental materials using SECT (Table 2). For materials containing smaller amounts of elements of a high atomic number and/or high densities (composites I and II, glass‐ceramic, and lithium disilicate), DE‐SACT‐based SPR prediction reached an SPR value of about 3.9 in most of the cases, leading to large deviations compared to measured SPR (Supplementary Table S3). This means that materials containing even small amounts of elements of a high atomic number and/or featuring high densities were overexpressed in the DECT reconstructions, except for glass‐ceramic, where possibly smaller amounts of a high atomic number element (yttrium) were used. These elements led to high CTN for these materials and thus an overestimation of their SPR values using a HLUT compared to measured SPR. For these same materials, DE‐DLCT‐based SPR reconstruction seemed to predict SPR more accurately, except for composite II (Table 2 and Supplementary Table S3). The larger SPR deviation in composite II could be due to the element ytterbium of a high atomic number, resulting in an SPR value of about 3.7 as for a metal alloy.

Furthermore, DE‐DirectSPR‐SACT and DE‐RhoZ‐SACT methods showed similar SPR predictions (Table 2). Since DE‐140‐SACT using image‐based methods relies on the 80 and 140 kV_p_ image datasets, it may be difficult to achieve reliable quantitative DECT data if CTN of one or both acquisitions are saturated or nearly saturated. For some materials (i.e., composites, glass‐ceramic, lithium disilicate), SPR prediction accuracy might seem better with SECT compared to DECT. This is mainly tied to the fact that the HLUT used in our institution and in this study has a maximum SPR value of 2.602 when using SE‐140‐SACT, which may be incidentally closer to the measured value. However, other institutions might use a HLUT with a maximum SPR value of approximately 4, which might then cause a larger overestimation of SPR values for dental materials saturating the CTN scale from the measured values. Moreover, we found that the insert diameter had an impact on SPR prediction accuracy. For example, cobalt‐chrome, composites, lithium disilicate, and zirconium dioxide didn't saturate the CTN scale anymore, when smaller insert diameters were fabricated and used. Thus, even smaller (more patient‐realistic) sizes of dental materials may result in better DECT‐based SPR predictions. DE‐RhoZ‐DLCT‐based SPR prediction with a mean deviation of 9.7% (range: [−35.43, 14.17]%) was consistently closer to measured SPR values compared to SE‐120‐DLCT showing a mean deviation of 23.9% (range: [−59.69, 44.25]%) (Table 2, excluding composite II). In contrast to human tissue, however, for dental materials containing elements of a high atomic number (e.g., ytterbium or zirconium), accurate SPR predictions remain still challenging with current DECT techniques. In particular, the accuracy of RED and EAN maps can reach its limit for specific materials consisting of elements of a high atomic number. Previous works reported that already a small trace of an element of a high atomic number causes a substantial positive bias in RED using DECT.^48^ As an example, a small amount of iodine in the thyroid was found to cause a positive bias in RED of about 1.1% for DE‐SACT and 0.3% for DE‐DLCT, which is most likely due to the larger influence of the photoelectric effect.^48^ An increasing amount of a high atomic number element also increases the bias in RED, although DE‐DLCT seems to be less affected than DE‐SACT, which was confirmed by our data (Table 2). Therefore, an SPR overestimation as observed for almost all dental materials containing elements of a high atomic number (Table 2) is an inherent property of the investigated DECT‐based SPR prediction methods as reported in previous studies.^16^, ^48^ Since the DE‐RhoZ and DE‐DirectSPR algorithms are implemented for the usual CTN range from −1024 HU to +3071 HU, an extended CTN scale doesn't provide additional information to improve SPR prediction. However, using an extended CTN scale may reveal more information about the dental material, including components and actual dimensions, that may be used to override the material with the proper SPR value. While current implementation does not seem to improve SPR prediction within dental materials, the use of MAR algorithms or virtual monoenergetic images may also reduce metal artifacts and improve structure delineation.^3^, ^30^ Altogether, DE‐SACT‐ and DE‐DLCT‐based SPR prediction provide improved SPR prediction accuracy compared to SECT for dental materials, especially for certain materials, for example, zirconium dioxide, and may improve treatment planning.

DECT‐based particle therapy treatment planning for head and neck cancer patients was evaluated for one exemplary dental material in an anthropomorphic head phantom. In the tooth surrogates (tooth dentin and tooth enamel) of the head phantom where no dental implant or restoration material is present, DECT‐based SPR predictions outperformed SECT (Table 3). SECT overestimated the SPR values of tooth dentin and tooth enamel compared to measured SPR values, whereas DECT slightly underestimated SPR values with deviations <1%. Since an SPR accuracy within 1% can be realistically reached using DECT in idealized situations,^11^ the DECT‐predicted SPR accuracy of the tooth surrogates was within the expected uncertainty. Moreover, the relative residuals of DECT‐predicted SPR values were in the same order of magnitude as the uncertainty of the measured SPR values of the tooth surrogates (0.2%).^16^ SECT‐predicted SPR values may have overestimated measured SPR values because the CTN of the tooth surrogates are relatively high due to their elemental compositions, which convert to higher SPR values using a HLUT than expected from the measurements.

Assessment of treatment planning for helium ion beams with the anthropomorphic head phantom containing dental materials revealed that SECT‐ and DECT‐based range predictions using SACT and DLCT were similar in the head phantom for beams unobstructed by dental material, respectively (Figure 5). Drawing on previous results from the same anthropomorphic head phantom using a validated ground truth SPR map,^16^ we assumed that DECT‐based SPR prediction is closer to the ground truth than SECT. However, an uncertainty of 1 mm has to be assumed due to the voxel size and from the manual delineation of the dental restoration. For the dental material, SECT‐ and DECT‐based SPR predictions were compared to a reference dataset, which is based on the measured SPR value of lithium disilicate and therefore as accurate as the SPR measurement itself. When ion beams passed through the dental material, DE‐SACT overestimated the SPR value of the lithium disilicate restoration with a relative deviation of about 35% compared to measured SPR (Table 3). Thus, current DE‐DirectSPR‐SACT SPR predictions may not be accurate enough for the specific material lithium disilicate at the moment. DE‐RhoZ‐DLCT with a relative deviation of 0.2% was closer to measured SPR than SE‐120‐DLCT (Table 3). Using projection‐based DE‐DLCT for patients with a lithium disilicate restoration showed better agreement with measured SPR than SECT (Figure 5) and may be used for future particle therapy treatment planning strategies.

In general, this study showed that, in its current state, image‐based spectral reconstruction as used with SACT may be limited in making quantitative statements for certain non‐tissue dental materials, especially if the materials contain elements of a high atomic number, for example, composite II with ytterbium as investigated in Section 3.2. Projection‐based spectral reconstruction may provide superior SPR prediction for some materials. However, the extent of improvement in dental management is largely dependent on the components, size, and shape of the material and therefore must be evaluated on a case‐by‐case basis to determine whether DECT‐based SPR prediction is currently accurate enough for particle therapy treatment planning. The department of prosthodontics could preferentially use dental implant and restoration materials that are more suitable for DECT‐based SPR prediction, if this is possible for the patient.

### 4.2 Comparison to previous work

This study found that both subjective image quality and SPR prediction accuracy decreased for dental materials with higher densities (cobalt‐chrome, composites, and zirconium dioxide). This is in line with previous works reporting that highly attenuating materials such as zirconium (*ρ* ≈ 6.5 g/cm^3^) or cobalt‐chrome (*ρ* ≈ 8.5 g/cm^3^) cause more severe artifacts on CT image datasets, resulting in larger artifact index and lower image criteria scores compared to the artifacts caused by titanium (*ρ* ≈ 4.5 g/cm^3^).^49^, ^50^

A previous study investigated a base metal, amalgam, lithium disilicate, and zirconia and observed substantial changes in proton range with respect to water for these materials.^20^ The present study confirmed that ion beam range was affected by the lithium disilicate restoration in the head phantom and that current SECT‐based SPR predictions don't provide sufficiently accurate range predictions in treatment planning (Table 3 and Figure 5). Compensating for the lithium disilicate restoration may be instead possible with projection‐based DE‐DLCT methods. For beams unobstructed by dental material in the anthropomorphic head phantom, the DE‐RhoZ‐SACT‐determined SPR values for the tooth dentin of 1.496 ± 0.019 and tooth enamel of 1.753 ± 0.020 were comparable to the SPR results of 1.496 ± 0.011 and 1.869 ± 0.018 from a previous study,^16^ although the SPR value for the dental enamel from this study was closer to the measured value.

### 4.3 Clinical relevance

This study focused on the radiological and dosimetric effects of dental materials that are common in an aging population receiving radiotherapy. Despite sophisticated imaging technology, dental material‐related effects on treatment planning remain a challenge in daily practice. In a previous study, a high incidence of over 70% of dental material artifacts was found on the planning CT image datasets of oral/oropharyngeal cancer patients.^51^ The finite range of ion beams makes them more sensitive to planning uncertainties than photon beams; therefore, it is especially important to improve dental management for particle therapy.^5^

To our knowledge, this is the first study that comprehensively investigated and characterized common state‐of‐the‐art dental implant and restoration materials using two different DECT techniques. To this end, radiological material parameters were determined and SPR values measured and compared between SECT and DECT techniques. For the investigated dental materials, measured SPR values can be assigned in treatment planning for patients with known, accurately contoured dental materials to account for uncertainties in SPR values. Moreover, robust optimization, using several CT image datasets (e.g., SECT, DECT, MAR, materials override…) of the patient and/or additional range uncertainty optimization parameters, may be applied to consider dental materials. In silico study using an anthropomorphic head phantom revealed that DE‐DLCT‐based SPR prediction for patients with lithium disilicate restorations may enable treatment planning despite the dental material. Thus, uncertainties in SPR prediction may be limitable and manageable using DECT.

### 4.4 Study limitations and future work

This study investigated a representative selection of common dental materials. However, no amalgam samples were investigated, because the institutional department of prosthodontics no longer uses amalgam as a filling material, but elderly patients may still harbor amalgam. Moreover, no gold alloy was considered due to cost reasons. Finally, this study investigated two different DECT techniques (SACT and DLCT); however, other DECT techniques (e.g., dual‐source CT, twin‐beam CT, or fast kVp‐switching CT) may change SPR prediction results. Using different dual‐energy tube voltage combinations (e.g., 100 and 140 kV_p_), for example, for SACT and dual‐source CT may also influence results, but was not possible to employ with our CT scanner settings.

High‐energy data acquired with photon‐counting detector CT systems has been shown to reduce metal artifacts in reconstructions owing to reduced beam hardening.^52^ With further development, photon‐counting CT may offer the potential for improved SPR prediction and dose calculation for patients with dental materials.

In the future, dental implant and restoration materials which are often composed of various components could be manufactured with a lower amount of radiopaque material, which could be explored by vendors. Nevertheless, it will still be necessary to distinguish non‐tissue dental materials from natural teeth in planar x‐ray imaging and CT image acquisitions. The discovery of new materials or compositions without elements of a high atomic number for usage in dentistry might reduce SPR prediction uncertainty in the future. Interdisciplinary collaborations are needed for the management of non‐tissue dental materials. Institutional departments of prosthodontics might select dental implant and restoration materials that are more suitable for SPR prediction with SECT and DECT―depending on the available and used CT technology in a radiation therapy department.

Finally, other non‐tissue implant materials in the body (e.g., spinal stabilization implants, hip prostheses, or silicone breast implants^53^) may also benefit from using DECT‐based SPR prediction. For example, by using DECT, the SPR overestimation for PALACOS^®^ R + G bone cement (Heraeus, Hanau, Germany) was reduced from 50% to less than 10%.^18^ Future work might investigate other non‐tissue implant materials in the human body using DECT imaging for particle therapy treatment planning.

## 5 CONCLUSIONS

This study investigated DECT‐based imaging for particle therapy treatment planning for patients with dental implant and restoration materials by using sequential acquisition and DLCT techniques. Radiological material parameters of ten common dental implant and restoration materials were determined. Overall, DECT‐based SPR predictions of cylindrical inserts in a geometric phantom showed better agreement with measured reference data compared to SECT‐based SPR predictions. DECT‐based helium ion therapy treatment planning in an anthropomorphic head phantom with dental material indicated that DE‐SACT and DE‐DLCT predicted similar ranges for beams unobstructed by dental material. When ion beams passed through the lithium disilicate restoration, DE‐DLCT‐based SPR prediction using a projection‐based method was closest to measured reference data resulting in a remaining range deviation of 0.2 mm. In sum, DECT‐based SPR prediction may improve the management of non‐tissue dental implant and restoration materials and subsequently compensate for them during particle therapy treatment planning. Further studies and interdisciplinary collaborations with departments of prosthodontics may assess other dental materials and techniques to further reduce SPR prediction uncertainty in dental materials. Prosthodontists stand to gain from such collaboration, for without it, particle therapy may require them to perform dental extractions for cancer patients with obstructive dental material,^5^ which will have to be removed and then replaced with an alternative. Ongoing collaboration, however, would help to identify optimal materials for both prosthodontics and radio‐oncology, thus incentivizing future research together to improve patient outcomes.

## AUTHOR CONTRIBUTIONS

*Conceptualization*: Friderike K. Longarino, Christopher Herpel, Wolfram Stiller, and Andrea Mairani. *Methodology*: Friderike K. Longarino, Christopher Herpel, Thomas Tessonnier, Wolfram Stiller, and Andrea Mairani. *Data acquisition and analysis*: Friderike K. Longarino, Thomas Tessonnier, and Benjamin Ackermann. *Interpretation*: Friderike K. Longarino, Thomas Tessonnier, Wolfram Stiller, and Andrea Mairani. *Writing—original draft preparation*: Friderike K. Longarino. *Writing—review and editing*: Friderike K. Longarino, Christopher Herpel, Thomas Tessonnier, Stewart Mein, Benjamin Ackermann, Jürgen Debus, Franz Sebastian Schwindling, Wolfram Stiller, and Andrea Mairani. *Supervision*: Jürgen Debus, Franz Sebastian Schwindling, Wolfram Stiller, and Andrea Mairani. All authors contributed to the article and approved the submitted version.

## CONFLICT OF INTEREST STATEMENT

The authors declare no conflicts of interest.

## Supporting information

Supporting Information
