# Supplementary material for: Dual‐energy CT‐based stopping power prediction for dental materials in particle therapy
Source: J Appl Clin Med Phys. 2023 Apr 9;24(8):e13977. doi: 10.1002/acm2.13977 (PMC10402687; doi:10.1002/acm2.13977)
Supplement: Supplementary file 1 — Supporting Information [file ACM2-24-e13977-s001.pdf]

Dual-energy CT-based stopping power prediction for dental materials in particle therapy

Supplementary Material

Supplementary Table 1. Dental materials investigated in the study with their main components, main application field, and manufacturing details.

Material type	Main components	Main application field	Manufacturing process	Product, Manufacturer
**Aluminum**	Al	Pure metal, component of aluminum oxide ceramics	CAD/CAM milling	Custom-made
**Cobalt-chrome**	CoCr	Fixed dental prostheses	CAD/CAM milling, casting	Colado CAD CoCr4, Ivoclar
**Composite I**	Mixture of dimethacrylates, silicates, ceramics, initiators, stabilizers	Core buildups, fillings	Manual layering and light-curing	Rebilda DC, VOCO
**Composite II**	Mixture of dimetracrylates, inorganic fillers, copolymer, ytterbium fluoride, initiators, stabilizers, pigments	Direct restorations, fillings	Manual layering and light-curing	Tetric EvoCeram, Ivoclar
**Glass-ceramic**	Mixture of leucite $KAlSi_2O_6$, fluorapatite $Ca_5(PO_4)_3F$, oxyapatite $NaY_9(SiO_4)_8O_2$	Veneers, inlays, partial crowns	Conventional layering	IPS Style Ceram, Ivoclar
**Lithium disilicate**	$Li_2O_5Si_2$	Fixed dental prostheses	CAD/CAM milling, press technology	IPS e.max Press, Ivoclar
**Polymethyl methacrylate (PMMA)**	$C_5H_8O_2$	Tissue retraction devices	3D-printing	V-print splint, VOCO
**Silicone material**	Mixture of silicone polymers and fillers with platinum catalyst	Tissue retraction devices	Dental impression taking	Silicone impression material: Flexitime Putty, Kulzer; Sealing silicone: Mucopren Silicone sealant, Kettenbach
**Titanium**	Ti	Dental implants	Casting, prefabricated	Gammex Electron Density CT Phantom 467, Gammex-RMI
**Zirconium dioxide**	ZrO_2	Fixed dental prostheses, dental implants	CAD/CAM milling	IPS e.max ZirCAD, Ivoclar

Supplementary Table 2. Image acquisition settings and reconstructions parameters of head protocols for sequential acquisition CT (SACT) and dual-layer spectral CT (DLCT). Tube current modulation was deactivated. Please note the different reference diameters for $CTDI_{vol}$ of the two protocols resulting from the different protocol setting options. For DLCT, an iDose[4] level of 0 was applied (Philips Healthcare, Best, The Netherlands), which corresponds to conventional filtered back-projection image reconstruction.

**Protocol**	**DECT technique**	**Tube voltage (kV_p)**	**Tube current-time product (mAs)**	**Collimation (mm)**	**Rotation time (s)**	**Pitch**	**$CTDI_{vol}$ (mGy)**	**Slice thickness and spacing (mm)**	**Reconstruction filter**
**Head**	SACT	80/140	247/58	2 x 32 x 0.6	0.5	0.55	11.1/12.7 (32 cm $CTDI_{vol}$ diameter)	1.5/1.5	Qr40f
**Head**	DLCT	120	300	64 x 0.625	0.5	0.8	47.2 (16 cm $CTDI_{vol}$ diameter)	1.5/1.5	UB

Supplementary Table 3. Dental materials investigated in the study with measured, single-energy CT (SECT)- and dual-energy CT (DECT)-predicted stopping power ratio (SPR) values using sequential acquisition CT (SACT) and dual-layer spectral CT (DLCT). No uncertainty (or 0) was reported for certain materials for SE-120-DLCT because the CTN were saturated, resulting in a maximum SPR value. SE-140-SACT: SECT-based SPR prediction with SACT at 140 kV_p; DE-DirectSPR-SACT: DECT-based SPR prediction with SACT using a DirectSPR implementation; DE-RhoZ-SACT: DECT-based SPR prediction with SACT using the RhoZ-method; SE-120-DLCT: SECT-based SPR prediction with DLCT at 120 kV_p; DE-RhoZ-DLCT: DECT-based SPR prediction with DLCT using the RhoZ-method.

**Material**	**Measured SPR**	**SACT**			**DLCT**	
		**SPR_{SE-140}**	**$SPR_{DE-DirectSPR}$**	**$SPR_{DE-RhoZ}$**	**SPR_{SE-120}**	**$SPR_{DE-RhoZ}$**
**Aluminum**	2.140 ± 0.002	2.074 ± 0.003	2.268 ± 0.003	2.203 ± 0.022	2.006 ± 0.008	2.222 ± 0.008
**Cobalt-chrome**	5.823 ± 0.002	2.581 ± 0.008	3.997 ± 0.010	3.985 ± 0.026	2.347 ± 0.000	3.760 ± 0.001
**Composite I**	1.627 ± 0.001	2.585 ± 0.004	3.885 ± 0.009	3.898 ± 0.006	2.347 ± 0.000	1.749 ± 0.077
**Composite II**	1.744 ± 0.002	2.579 ± 0.003	3.971 ± 0.010	3.990 ± 0.006	2.347 ± 0.000	3.722 ± 0.015
**Glass-ceramic**	2.079 ± 0.002	2.384 ± 0.006	3.242 ± 0.006	3.251 ± 0.006	2.347 ± 0.000	2.155 ± 0.004
**Lithium disilicate**	2.091 ± 0.001	2.601 ± 0.001	3.968 ± 0.052	3.961 ± 0.070	2.347 ± 0.000	2.332 ± 0.009
**PMMA**	1.169 ± 0.001	1.092 ± 0.001	1.175 ± 0.003	1.177 ± 0.005	1.088 ± 0.002	1.176 ± 0.002
**Silicone material**	1.229 ± 0.003	1.242 ± 0.001	1.254 ± 0.007	1.257 ± 0.004	1.242 ± 0.003	1.246 ± 0.002
**Titanium**	3.248 ± 0.002	2.601 ± 0.001	3.878 ± 0.005	3.863 ± 0.008	2.347 ± 0.000	3.708 ± 0.061
**Zirconium dioxide**	4.160 ± 0.001	2.577 ± 0.007	4.022 ± 0.005	4.018 ± 0.033	2.347 ± 0.000	3.761 ± 0.001
